# Influences of Dementia on Long-Term Surgical Outcomes in Older Adults After Hip Fracture

**DOI:** 10.1093/geroni/igaa057.3246

**Published:** 2020-12-16

**Authors:** Tingzhong (Michelle) Xue, Eleanor McConnell, Chiyoung Lee, Hideyo Tsumura, Sijia Wei, Wei Pan

**Affiliations:** 1 Duke University, Durham, North Carolina, United States; 2 Durham, North Carolina, United States; 3 Duke University School of Nursing, Durham, North Carolina, United States

## Abstract

Older adults with dementia are more prone to have adverse health outcomes following hip fracture surgery. However, individuals with dementia and hip fracture are older and have more co-morbidities; these baseline differences can bias estimates of the influence of dementia. This study aims to investigate how dementia influences disposition, mortality rates and readmission rates at 365 days after hip surgery in older adults over age 65, after accounting for baseline factors such as socioeconomic status, health behaviors, co-morbidities, and type of hip fracture repair. A cohort of 1172 patients who had hip fracture surgery between October 2015 and December 2018 was extracted from electronic health records; among those, 376 had a diagnosis of dementia. Inverse probability of treatment weighting using propensity scores method was used to reduce the influence of factors that may confound the relationship between dementia status and hip surgery outcomes. Logistic regression was applied to estimate influences on discharge disposition and Cox proportional hazards model for one-year mortality. To account for competing risk of death, a Fine and Gray regression model was used to calculate subdistribution hazard ratios of readmission. Disparities in long-term surgical outcomes in patients with dementia were found. Results show that dementia was a significant predictor for being discharged to facilities (OR=1.92, 95% CI 1.09, 3.39, p=.025), death (HR=1.98, 95% CI 1.50-2.62, p<.0001) and being readmitted within one year (HR=1.31, 95% CI 1.15-1.50, p<.0001). These findings call for more efforts in developing effective multidisciplinary perioperative assessments and rehabilitation for patients with dementia.

